# Validation of Smartphone Based Retinal Photography for Diabetic Retinopathy Screening

**DOI:** 10.1371/journal.pone.0138285

**Published:** 2015-09-24

**Authors:** Ramachandran Rajalakshmi, Subramanian Arulmalar, Manoharan Usha, Vijayaraghavan Prathiba, Khaji Syed Kareemuddin, Ranjit Mohan Anjana, Viswanathan Mohan

**Affiliations:** Madras Diabetes Research Foundation & Dr.Mohan’s Diabetes Specialities Centre, WHO Collaborating Centre for Non-communicable Diseases Prevention and Control, IDF Centre of Education, Gopalapuram, Chennai, India; Univ Rochester Medical Ctr, UNITED STATES

## Abstract

**Aim:**

To evaluate the sensitivity and specificity of “fundus on phone’ (FOP) camera, a smartphone based retinal imaging system, as a screening tool for diabetic retinopathy (DR) detection and DR severity in comparison with 7-standard field digital retinal photography.

**Design:**

Single-site, prospective, comparative, instrument validation study.

**Methods:**

301 patients (602 eyes) with type 2 diabetes underwent standard seven-field digital fundus photography with both Carl Zeiss fundus camera and indigenous FOP at a tertiary care diabetes centre in South India. Grading of DR was performed by two independent retina specialists using modified Early Treatment of Diabetic Retinopathy Study grading system. Sight threatening DR (STDR) was defined by the presence of proliferative DR(PDR) or diabetic macular edema. The sensitivity, specificity and image quality were assessed.

**Results:**

The mean age of the participants was 53.5 ±9.6 years and mean duration of diabetes 12.5±7.3 years. The Zeiss camera showed that 43.9% had non-proliferative DR(NPDR) and 15.3% had PDR while the FOP camera showed that 40.2% had NPDR and 15.3% had PDR. The sensitivity and specificity for detecting any DR by FOP was 92.7% (95%CI 87.8–96.1) and 98.4% (95%CI 94.3–99.8) respectively and the kappa (ĸ) agreement was 0.90 (95%CI-0.85–0.95 p<0.001) while for STDR, the sensitivity was 87.9% (95%CI 83.2–92.9), specificity 94.9% (95%CI 89.7–98.2) and ĸ agreement was 0.80 (95%CI 0.71–0.89 p<0.001), compared to conventional photography.

**Conclusion:**

Retinal photography using FOP camera is effective for screening and diagnosis of DR and STDR with high sensitivity and specificity and has substantial agreement with conventional retinal photography.

## Introduction

The increasing global burden of diabetes has resulted in an increase in the prevalence of both microvascular and macrovascular complications of diabetes [1]. Early detection and treatment of the complications of diabetes are the cornerstones to reduce morbidity due to diabetes [2]. Diabetic retinopathy (DR) is one of the important causes for blindness in adults with diabetes and its prevalence is high in most parts of the world [3]. Thus visual impairment due to diabetic retinopathy remains a significant health burden.

The value of screening for diabetic retinopathy (DR) is well established as majority of the patients who develop DR have no symptoms until visual impairment occurs due to the sight threatening stages of DR namely severe diabetic macular edema (DME) and/or proliferative diabetic retinopathy (PDR) [4,5]. DR has a fairly long asymptomatic stage, during which, it can be easily identified by fundus examination or retinal photography.

India is home to over 65 million people with diabetes [6]with an estimated DR prevalence of 18% [7]. Hence, as per the ADA guidelines [8], over 65 million Indians with diabetes need to be screened for DR every year.Dilated fundus examination using direct and indirect ophthalmoscopy by a trained ophthalmologist, is the ideal screening method for DR [6]. Many studies have suggested that retinal photography after dilatation is equivalent or superior to ophthalmoscopic examination for detection of DR [9,10].

Mydriatic seven field stereoscopic retinal color photography remains the gold standard method for screening of DR (ETDRS) [11]. However, digital retinal photography and nonmydriatic fundus photography [12] are more practical alternatives for screening people with diabetes for retinopathy. The limiting factors for screening large numbers of people with diabetes include lack of adequate number of ophthalmologists/ retina specialists and/or trained eye technicians/ optometrists as well as the non-availability and high cost of the conventional fundus cameras [13–15].

This study utilizes a novel, indigenous, sleek smart phone based device for retinal colour photography which can be used for screening of DR both in the clinic and well as in tele-ophthalmology.

The objective of this paperis to validate retinal imaging by ‘fundus on phone’ camera against the conventional 7 field digital fundus photography using a high end fundus camera, both for screening of DR as well as detect DR of varying severity.

## Methods

Three hundred and one consecutive known diabetic patients with varying duration of diabetes referred to the Eye Department of a large network of tertiary care hospitals for diabetes in Chennai (formerly Madras) in southern India were invited to participate in this prospective instrument validation study. The sample size for this study was calculated based on a pilot study conducted with 50 patients for assessing the sensitivity and specificity of FOP, to detect DR. The inclusion criteria for patients were age more than 18 years, type 2 diabetes, no contraindication to mydriasis, no allergy to tropicamide eye drops and willingness to undergo mydriatic retinal colour photography with two fundus cameras. A written informed consent was obtained from all the participants and the study was conducted over a period of 3 months (Dec 2014–Feb 2015) after the approval of the Institutional Ethics Committee.

All patients underwent a complete detailed ocular examination. A preliminary eye examination, which included a visual acuity test using an illuminated Snellen vision chart, intra-ocular pressure measurement by non-contact tonometry and a slit lamp examination of the anterior segment of the eye to assess the depth of anterior chamber and presence of media opacities like cataract was done. Dilatation was done by administration of tropicamide eye drops.

Mydriatic seven-field digital retinal color photography was first taken by a trained photographer/ optometrist using the Zeiss FF450 Plus Digital Fundus Camera (Carl Zeiss Meditec, Inc. Dublin, CA). The 7 fields photographed were the macula, optic disc, superior-temporal, superior nasal, inferior nasal, inferior-temporal and temporal macula fields of each eye.

**Fig 1:**
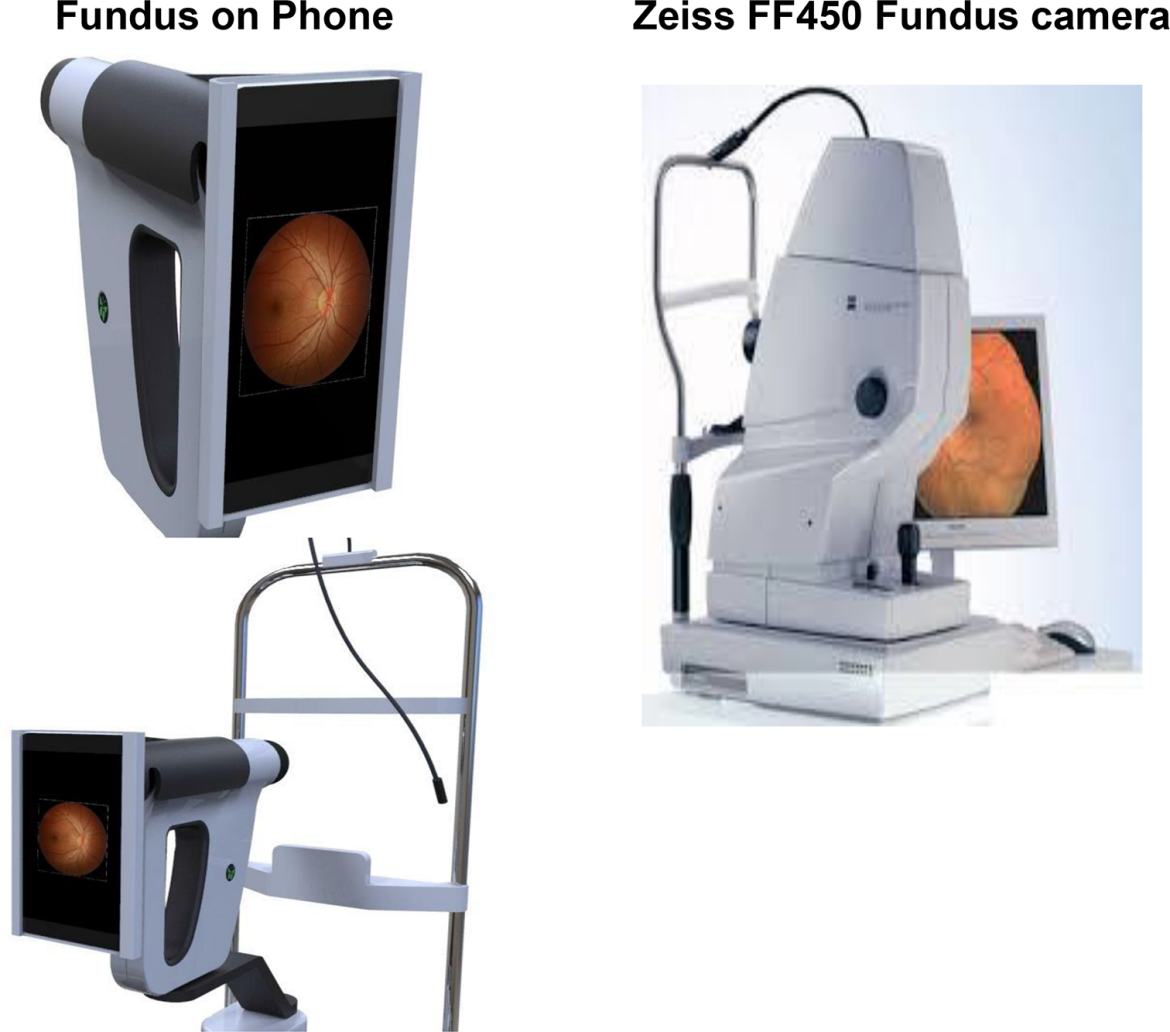
Fundus cameras.

The participants then underwent a 4 field retinal colour photography using the “Fundus on Phone” (FOP) smartphone based retinal imaging system (Remidio Innovative Solutions Pvt Ltd, Bangalore). The fields which were captured on the FOP camera were macula, disc and nasal to the optic disc, superior-temporal and inferior-temporal quadrants [7]. The FOP is a portable retinal camera capable of being used in both clinical set-up and in field settings. It consists of an annular illumination optical design which mates with a commercially available smart phone to acquire and transmit retinal images **(Fig 1)**. The illumination design ensures reflex-free images of the retina, in a compact format, and it is much smaller than the traditional fundus cameras **(Fig 1).** FOP has a 45 degree field of view, a 33 mm working distance, +20 to -20 dioptre adjustment and an optical magnification of 12X. The sensor resolution is governed by the smart phone. The device can be fit onto any standard slit lamp as shown in **Fig 1**. The auto-focus of the camera app, allows for sharp images of the retina to be taken, using the smartphone’s touch interface. An android app allows all data of the patient to be stored folder wise on the phone enabling easy archival and retrieval. Each photograph has patient data in the corner to prevent any mix ups during the acquisition, transfer and reporting process. Unlike traditional fundus cameras, that operate using a xenon flash, the illumination on FOP uses a warm-white LED that has a life of more than 50,000 hrs. However the device does not have a mechanical tilt, as is typically available in the Zeiss fundus camera.

Photographs were coded using an identification number and assessed in a masked manner for the presence and severity of DR in order to minimize any possible bias. The photographs were graded separately by 2 ophthalmologists (retina specialists) (SA and MU), who were masked to the clinical diagnosis. Disagreement in the retinopathy grading between the two graders was adjudicated by a 3^rd^ retina specialist (RR). The grading of retinopathy was done based on the modified ETDRS grading system [11]. The minimum criterion for diagnosis of DR was the presence of at least one definite microaneurysm in any field of the retina [7]. Each eye was graded separately and the retinal photographs were assessed and assigned a retinopathy level. The final diagnosis for each patient was determined from the level of DR of the worse eye using ETDRS retinopathy scale (Level 10 represents no retinopathy, level 20 to 53 non-proliferative diabetic retinopathy [NPDR], level ≥60, proliferative diabetic retinopathy [PDR]) [11].Diabetic macular edema (DME)was defined as the presence of definite hard exudates within one disc diameter of the centre of the macula [16].DME may be present in all stages of retinopathy (NPDR or PDR). Sight-threatening diabetic retinopathy (STDR) was defined as PDR or DME (clinically significant macular edema) in either or both eyes [5,17].

### Image quality

The photographs taken in both the cameras were graded on a 5 level grading scheme as follows. Grade 0- ungradable (No detail was visible due to media opacities like dense cataract or total vitreous hemorrhage) [18], Grade 1- poor (Only gross retinal details, larger lesions like blot hemorrhages and dense hard exudates were detectable), Grade 2- average (Major retinopathy details visible; minor degrees of retinopathy like microaneurysms, intra-retinal microvascular abnormalities (IRMA) or subtle new vessels not clearly detectable), Grade 3 –good (Retinal details fairly clear, most of retinopathy changes detectable), Grade 4- excellent (all retinal details and retinopathy lesions clearly visible).

### Statistical analysis

All statistical analyses were performed using SPSS statistical package version 15.0. Continuous data are expressed as mean ± standard deviation while categorical data are presented as proportions.The sensitivity and specificity for detecting DR and diagnosing DR of varying degrees of severity were calculated for the FOP taking the dilated, 7-field fundus photography by the traditional Zeiss fundus camera as the gold standard. The degree of agreement between FOP and gold standard Zeiss 7-field fundus photography was quantified and assessed using kappa (ĸ) statistics. For all statistical tests, p value <0.05 was considered significant.

## Results

Three hundred and one known diabetic patients (602 eyes) completed both modes of retinal photography. 194 (64.5%) patients were males. The mean age of the participants was 53.5 ± 9.6 years (range: 20 years to 77 years) and the mean duration of diabetes was 12.5 ± 7.3 years (range: 1 day to 34.6 years of disease duration). None of the images were ungradable in the 602 eyes.

**Fig 2:**
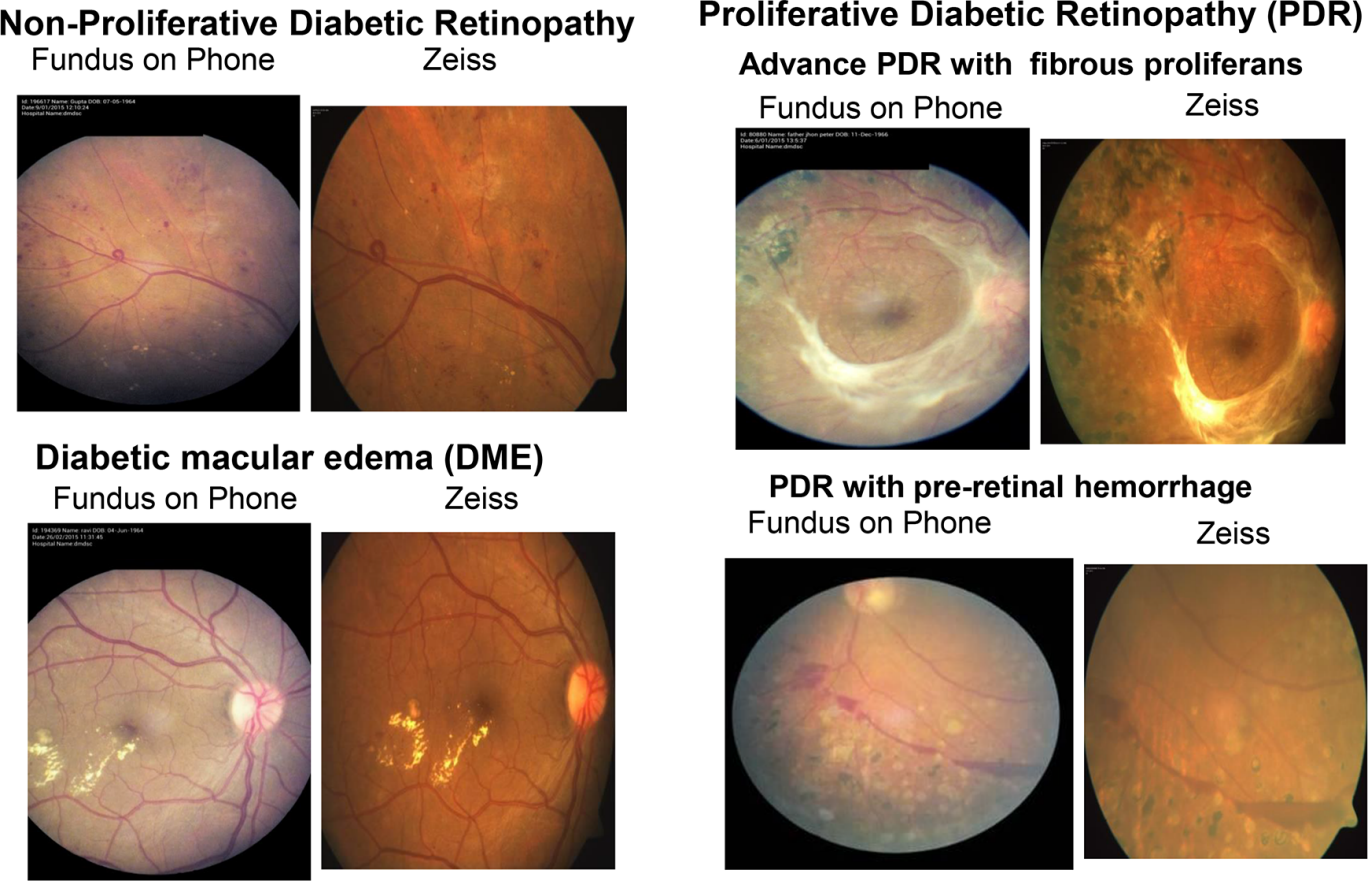
Retinal images of diabetic retinopathy obtained in fundus on phone (FOP) and Zeiss camera.

The overall prevalence of DR based on retinal photography by Zeiss fundus camera and FOP camera was 59.1% and 55.5% respectively. The DR detection matched in 165 out of 178 (92.7%) patients. Conventional fundus photograph showed that 43.9% had non-proliferative DR (NPDR) and 15.3% had PDR while the FOP camera showed that 40.2% had NPDR and 15.3% had PDR. The varying grades of diabetic retinopathy in the two modes of retinal photography are depicted in **Table 1.** DME was present in 82 (27.2%) patients based on the grading in both cameras. The agreement matched between the 2 cameras in 71 (86.6%) patients with DME. **Fig 2**shows the retinal images of varying degrees of severity of DR taken on FOP and Zeiss fundus camera.

**Table 1:** Diabetic retinopathy (DR) severity based on Fundus on phone (FOP) and Zeiss retinal photography

FOP CAMERA			ZEISS CAMERA			
	No DR n (%)	Mild NPDR n (%)	Moderate NPDR n (%)	Severe NPDR n (%)	PDR n (%)	Total
** No DR n (%)**	121 (40.2)	9 (3.0)	4 (1.3)	0	0	134 (44.5)
**Mild NPDR n (%)**	1 (0.3)	29 (9.7)	6 (2.0)	2(0.7)	1 (0.3)	39 (13.0)
**Moderate NPDR n (%)**	1 (0.3)	8 (2.7)	43 (14.3)	5 (1.7)	1 (0.3)	58 (19.3)
**Severe NPDR n (%)**	0	1 (0.3)	7 (2.3)	14 (4.7)	2 (0.7)	24 (8)
** PDR n (%)**	0	0	3 (1.0)	2 (0.7)	41 (13.6)	46 (15.3)
** TOTA**L	123 (40.9)	47(15.6)	63 (20.9)	22(7.3)	46 (15.3)	301 (100)

DR- Diabetic retinopathy

NPDR- Non-proliferative diabetic retinopathy

PDR—Proliferative diabetic retinopathy.

The sensitivity and specificity for detecting any DR and varying severity of DR by FOP against the Zeiss camera and the degree of agreement between the 2 fundus cameras are shown in **Table 2.** The degree of agreement between FOP and Zeiss fundus camera for any DR was 0.90 (95% CI- 0.85, 0.95 p<0.001) using the ĸ statistics. The ĸ agreement for the NPDR, PDR, DME and STDR groups was also similarly very good (**Table 2**).

**Table 2:** Sensitivity and specificity for diabetic retinopathy detection by retinal photography on fundus on phone (FOP) camera in comparison to Zeiss camera and the kappa agreement.

Status of Retinopathy	Sensitivity % (95%CI)	Specificity % (95% CI)	ĸ agreement (95% CI)	p value
**Any DR**	92.7% (87.8–96.1)	98.4% (94.3–99.8)	0.90 (0.85–0.95)	<0.001
**NPDR**	89.8% (83.3–94.4)	98.4%(94.3–99.8)	0.87 (0.82–0.92)	<0.001
**PDR**	89.1% (85.4–95.5)	95.8%(90.5–98.6)	0.84 (0.78–0.90)	<0.001
**DME**	86.6% (79.9–92.3)	94.6% (89.8–97.4)	0.79(0.72–0.88)	<0.001
**STDR**	87.9% (83.2–92.9)	94.9% (89.7–98.2)	0.80 (0.71–0.89)	<0.001

DR- Diabetic retinopathy

NPDR- Non-proliferative diabetic retinopathy; PDR- Proliferative diabetic retinopathy

DME- Diabetic macular edema, STDR- Sight threatening diabetic retinopathy.

The image quality of both the cameras, were also compared and graded by the retina specialists. None of the images taken in the 2 cameras were ungradable (grade 0). The image quality of retinal photographs taken in Zeiss was average (grade 2) in 34.9%, good (grade 3) in 53.5% and excellent in 7.6% (grade 4) while in FOP camera, the quality of retinal photographs was average (grade 2) in 66.8%, good (grade 3) in 17.9% and excellent in 4% (grade 4). The image quality of Zeiss was better than the FOP system.

## Discussion

Screening for diabetic retinopathy is highly cost effective as with early detection, DR can be treated by various methods that can preserve vision and reduce the risk of visual morbidity. Screening for DR by retinal colour photography has the advantage of being faster with easier acquisition, storage and transmission of retinal images. A cost effective sensitive retinal imaging system is needed in most middle and lower income countries for regular DR screening [19].

This is the first study to the best of our knowledge that has evaluated and validated an indigenous low cost fundus on phone camera to detect and grade diabetic retinopathy in comparison to retinal photographs taken by a conventional mydriatic fundus camera.

The effectiveness of any screening tool is assessed by its sensitivity and specificity. The sensitivity and specificity of retinal imaging using FOP for any DR was very high (92.7% and 98.4%) and for STDR was also high (87.9% and 94.9%) respectively. In a recent publication by Russo et al [20], comparing smartphone photography using an adapter (D Eye) and slitlamp biomicroscopy for grading of DR, the kappa agreement was 0.78. In our study the kappa agreement was higher at 0.90 for any DR. The kappa agreement for DME in the Russo study [20] and our study are similar. Mydriatic fundus photography which provides reasonably good image quality could be one of the reasons for the high sensitivity and specificity and a substantial degree of agreement between cameras in this study.

Nonmydriatic photography has been used as a practical DR screening alternative, especially in teleophthalmology [12,15,21,22]. A recent publication by Gupta et al [21], done to evaluate the sensitivity and specificity of non-mydriatic retinal imaging as a screening tool to detect DR in Indian eyes showed a low sensitivity and specificity for detecting any DR (58.8% and 69.1%) and STDR (63.1% and 68.9%). Factors such as longer duration of diabetes causing poor mydriasis, darker iris in Indians, and older age with media opacities like cataract, affects the image quality and hence can decrease the sensitivity and specificity of DR screening in non-mydriatic retinal imaging [21, 23]. The mean age of the patients is relatively lower in our study, which is probably one of the possible reasons for higher sensitivity and specificity for DR screening in the study.

In a study done by Scanlon et al [24], comparing the sensitivity and specificity of mydriatic and nonmydriatic digital retinal screening, with dilated slit lamp biomicroscopy as the reference standard, it was shown that mydriatic digital photography was an effective method of screening for DR while non-mydriatic fundus photography had an unacceptable technical failure rate and low specificity. Murgatroyd et al evaluated DR screening with a non-mydriatic camera and found that mydriasis was found to reduce the proportion of ungradable photographs from 26% to 5% [25]. In our study, there were no ungradable images in the fundus photographs taken with either of the cameras as none of the recruited patients had very dense cataract or total vitreous hemorrhage [18, 23]. When compared to Zeiss, the clarity of FOP images was poor in some patients due to hazy media and poor mydriasis and in others possibly due to lack of proper focussing. Hence some mild DME lesions and some subtle new vessels in the periphery in PDR were missed in the FOP images. Although the image quality was better with the Zeiss camera, the FOP also had reasonably good quality gradable images. This emphasises that mydriasis should not be considered as a disadvantage in DR screening.

The FOP has several advantages. The time taken for retinal imaging with FOP is less than 1 minute for each eye and the autofocus in FOP helps to obtain sharp focus and good quality retinal images. The option of zooming on the smartphone touch screen to visualise and enlarge images instantly and capture specific retinal lesions is an added advantage. The comfort of the participants while retinal images were taken with FOP was better than Zeiss as the light intensity of the LED light was lower, and hence there was no discomfort due to high intensity flash.

Moreover, the FOP retinal imaging device is significantly cheaper than the conventional mydriatic fundus cameras as well as the non-mydriatic fundus cameras currently used in tele-ophthalmology. The FOP is priced in India, at approximately one-fifth the price of a good non-mydriatic fundus camera and to one-tenth to one-fifteenth the price of a high quality conventional mydriatic camera.The use of long-life LED illumination and lithium ion battery in FOP as against xenon flash lamps and the need for uninterrupted power supply in conventional mydriatic camera reduce the operational cost of FOP. The use of the camera of consumer mobile phones and a smartphone application based image archival and retrieval system using mobile networks, versus high end original equipment manufacturer (OEM) sensors in traditional high end mydriatic cameras, helps keep the cost of the equipment, running cost and cost of spares lower in FOP. Annual maintenance contracts for the FOP system are priced at between one-sixth and one-twelfth of that of traditional retinal imaging systems.

The easy acquisition and storage of fundus images in separate folders, which can be directly transmitted from the smartphone via the wireless telecommunication system for remote evaluation, subsequent review and assessment by experts to determine the need for referral, are additional advantages of the FOP system. The sleekness, easy portability and the wireless connectivity with good battery life make it easy to be used in non-hospital settings for screening for DR in community outreach programmes.

In the recent past, smart phones have started becoming valuable diagnostic tools in the field of ophthalmology due to the availability of apps for assessment of visual acuity and the ability to perform fundus photography [26,27]. Many new smartphone adapters like Peek (Portable Eye Examination Kit by Stewart Jordan et al, United Kingdom) and Eye Go (Stanford School of Medicine) have been recently devised with the aim of providing eye examination and retinal photography in remote areas. These adapters along with the smartphone to which they will be attached, offer the functionality of a retinal camera. However neither of these devices has been validated by a study for screening for diabetic retinopathy. All the current smartphone camera based retinal photography require mydriasis.

Our study has some limitations. It is a study done in a single hospital in a clinic setting. Therefore the reported results cannot be directly transposed to a field setting. Future prospective studies in the field setting will help to validate its use in tele-ophthalmology for DR screening. The quality of the images of FOP system was not as good as that obtained in the conventional Zeiss camera; however majority (85%) of the images were average to good in the FOP as well.

In conclusion, this study shows that a smartphone based retinal imaging system, the fundus on phone is reasonably sensitive and specific in detecting diabetic retinopathy of varying severity and can be tried as an effective screening tool for diabetic retinopathy. The combination of affordability, portability, easy transmission of images and other features of this fundus on phone system provide a platform not only for in-clinic use but also for planning mass DR screening programs in India and other low and middle income countries.
